# Diversity in bacterium-host interactions within the species *Helicobacter heilmannii* sensu stricto

**DOI:** 10.1186/1297-9716-44-65

**Published:** 2013-07-29

**Authors:** Myrthe Joosten, Caroline Blaecher, Bram Flahou, Richard Ducatelle, Freddy Haesebrouck, Annemieke Smet

**Affiliations:** 1 Department of Pathology, Bacteriology and Avian Diseases, Faculty of Veterinary Medicine, Ghent University, Salisburylaan 133, Merelbeke 9820, Belgium

## Abstract

*Helicobacter (H.) heilmannii* sensu stricto (s.s.) is a zoonotic bacterium that naturally colonizes the stomach of dogs and cats. In humans, this microorganism has been associated with gastritis, peptic ulcer disease and mucosa associated lymphoid tissue (MALT) lymphoma. Little information is available about the pathogenesis of *H. heilmannii* s.s. infections in humans and it is unknown whether differences in virulence exist within this species. Therefore, a Mongolian gerbil model was used to study bacterium-host interactions of 9 *H. heilmannii* s.s. strains. The colonization ability of the strains, the intensity of gastritis and gene expression of various inflammatory cytokines in the stomach were determined at 9 weeks after experimental infection. The induction of an antrum-dominant chronic active gastritis with formation of lymphocytic aggregates was shown for 7 strains. High-level antral colonization was seen for 4 strains, while colonization of 4 other strains was more restricted and one strain was not detected in the stomach at 9 weeks post infection. All strains inducing a chronic active gastritis caused an up-regulation of the pro-inflammatory cytokine IL-1β in the antrum. A reduced antral expression of H^+^/K^+^ ATPase was seen in the stomach after infection with 3 highly colonizing strains and 2 highly colonizing strains caused an increased gastrin expression in the fundus. In none of the *H. heilmannii* s.s.-infected groups, IFN-γ expression was up-regulated. This study demonstrates diversity in bacterium-host interactions within the species *H. heilmannii* s.s. and that the pathogenesis of gastric infections with this microorganism is not identical to that of an *H. pylori* infection.

## Introduction

*Helicobacter (H.) pylori* is the most prevalent *Helicobacter* species colonizing the gastric mucosa of humans and has been associated with gastritis, peptic ulcer disease and gastric cancer [1-3]. Besides *H. pylori*, other morphologically distinct non-*H. pylori Helicobacter* (NHPH) species, also referred to as *H. heilmannii* sensu lato (s.l.) [4] have been associated with gastric disease in humans [5-10]. NHPH represents a group of closely related but distinct bacterial species, mainly found in different animal species, such as *H. felis*, *H. salomonis*, *H. bizzozeronii*, *H. heilmannii* sensu stricto (s.s.), *H. cynogastricus* and *H. baculiformis* in cats and dogs and *H. suis* in pigs [5,7,10-15]. These microorganisms are characterized by their extremely fastidious nature, which so far has resulted in a limited number of in vitro isolates available worldwide.

*H. heilmannii* s.s. has only recently been isolated and cultured in vitro [16]. It has been detected in wild feline and in human gastric biopsies, but it is most commonly found in the gastric mucosa of cats and dogs with a prevalence ranging from 20 to 100% [5-7,10,12]. Although this bacterium has been associated with chronic active gastritis in cats and dogs [12], its pathogenic significance remains enigmatic and is probably strain-dependent. In humans, *H. heilmannii* s.s. has been detected in 8–19% of gastric biopsies with histological evidence of NHPH infection [5,8,10]. Infection with this bacterium in humans has been associated with gastritis, peptic ulcer disease and mucosa associated lymphoid tissue (MALT) lymphoma [5,8,10,17,18].

Several infection studies in experimental animal models have been performed to investigate the pathogenesis of *H. pylori* infections in humans. In contrast, little information is available dealing with the pathogenesis of *H. heilmannii* s.s. infections in humans. This bacterium has been propagated in mice for up to 28 months and was able to induce MALT lymphoma in the stomachs of these animals [18]. However in this mouse experiment, homogenized gastric tissue was used as inoculum. This implies that other microorganisms were inoculated together with *H. heilmannii* s.s., which might influence the results, as has been described previously [19]. Thus, to obtain better insights into the pathogenesis of human gastric disease associated with *H. heilmannii* s.s., experimental infection studies with pure cultures of this microorganism are essential. Therefore, the aim of the present study was to study bacterium-host interactions of 9 *H. heilmannii* s.s. strains, isolated from the gastric mucosa of different cats. The Mongolian gerbil model has previously been shown to be a useful animal model to study *Helicobacter*-related gastric pathology in humans and was therefore used in the present study [19-21].

## Material and methods

### Bacterial strains

Nine strains of *H. heilmannii* s.s. were obtained from the gastric mucosa of different cats and designated ASB1 (= type strain, DSM 23983, [16]), ASB2, ASB3, ASB6, ASB7, ASB9, ASB11, ASB13 and ASB14. Bacteria were cultivated on biphasic *Brucella* agar plates (Oxoid, Basingstoke, UK) supplemented with 20% (v/v) fetal calf serum (HyClone, Logan, UT, USA), 5 mg/L amphothericin B (Fungizone, Brystal-Myers Squibb, New York, USA), Skirrow (Oxoid, contains 10 mg/L vancomycin, 5 mg/L trimethoprim lactate and 2500 U/L polymyxin B), Vitox supplement (Oxoid) and 0.05% HCl (pH 5). After incubation under microaerobic conditions (85% N_2_, 10% CO_2_, 5% O_2_; 37 °C), the bacteria were harvested and the final concentration was adjusted to 7 × 10^8^ viable bacteria/mL, as determined by counting in a Neubauer counting chamber.

### Animals, housing and experimental procedure

Specific-pathogen-free (SPF) female five-week-old Mongolian gerbils (Crl:MON (Tum), *n* = 48) were obtained from Charles River Laboratories (Lille, France). The animals were housed in filter top cages (1500 cm^2^) on autoclaved wood shavings and autoclaved hay. They were fed ad libitum an autoclaved commercial diet (TEKLAD 2018S, containing 18% protein; Harlan, The Netherlands) and autoclaved water. For each of the 9 *H. heilmannii* s.s. strains tested, 5 animals were intragastrically inoculated 3 times at 2 days interval with 300 μL of a bacterial suspension. Three animals were inoculated with *Brucella* broth (pH 5, Oxoid) and served as negative controls. Inoculation was performed under brief isoflurane anaesthesia (2.5%), using a ball-tipped gavage needle. At 9 weeks after the first inoculation, the animals were euthanized by cervical dislocation under deep isoflurane anaesthesia (5%). The stomach and the duodenum of each gerbil were resected and samples were taken for histopathological examination and quantitative real-time (RT)-PCR analysis.

The in vivo experiment was approved by the Ethical Committee of the Faculty of Veterinary Medicine, Ghent University, Belgium (EC 2011/090).

### Histopathology and immunohistochemistry

A longitudinal section, starting from the end of the forestomach and comprising the antrum and the fundus of the stomach and part of the duodenum, was cut along the greater curvature and fixed in 10% phosphate buffered formalin, processed by standard methods and embedded in paraffin for light microscopy. Three consecutive sections of 5 μm were cut. After deparaffinization and hydration, heat-induced antigen retrieval was performed in citrate buffer (pH 6). To block endogenous peroxidase activity and non-specific reactions, slides were incubated with 3% H_2_O_2_ in methanol (5 min) and 30% goat serum (30 min), respectively. The first section was stained with haematoxylin/eosin (H&E) to score the intensity of the gastritis according to the Updated Sydney System [22] but with some modifications, as described previously [19]. On the second section, epithelial cell proliferation was determined by immunohistochemical staining using a mouse monoclonal anti-Ki67 antibody (1/25; Menarini Diagnostics, Zaventem, Belgium). Ki67-positive epithelial cells were counted in 5 randomly chosen High Power Fields at the level of the gastric pits (magnification: 400×), both in antrum and fundus. The average of the positive cell count was calculated for each experimental group in both stomach regions.

Parietal cells were identified on the third section by immunohistochemical staining for the hydrogen potassium ATPase using a mouse monoclonal antibody (1/200; Abcam Ltd, Cambridge, UK). Incubation with primary antibodies directed against Ki67 and hydrogen potassium ATPase was followed by incubation with a HRP-labeled secondary antibody (Envision Link Mouse K4007, DakoCytomation, Heverlee, Belgium) for visualization.

### DNA extraction and quantification of colonizing *H. heilmannii* s.s. in the stomach and duodenum

From each gerbil, samples from the fundus and the antrum of the stomach and from the duodenum were taken. Tissue samples were stored in 1 mL RNA later (Ambion, Austin, TE, USA) at -70 °C until RNA- and DNA-extraction. Tissue samples were homogenized (MagNALyser, Roche, Mannheim, Germany) and RNA and DNA were separated using TriReagent RT (Molecular Research Center Inc, Cincinnati, USA) according to the manufacturer’s instructions. The number of colonizing *H. heilmannii* s.s. per mg gastric tissue was determined in the DNA samples using a *H. heilmannii* s.s.-specific quantitative RT-PCR. For generation of the standard, part of the *ureAB* gene cluster (1224 bp) from *H. heilmannii* s.s. ASB1 was amplified using primers U430F and U1735R, as described previously [6]. The standard consisted of 10-fold-dilutions starting at 10^8^ PCR amplicons for each 10 μL of reaction mixture. One μL of extracted DNA template was suspended in a 10 μL reaction mixture consisting of 0.25 μL of both primers located within the 1224 bp fragment, to yield a 212 bp PCR product (sense primer: HH_SP1: 5’-CTT TCT CCT GGT GAA GTG ATT CTC-3’ , antisense primer: HH_RVQ: 5’-GCT GTA CCA GAG GCA ATG TCC AAG-3’ , annealing temperature 58 °C), 3.5 μL HPLC water and 5 μL SensiMix™ SYBR No-ROX (Bioline Reagents Ltd, UK). Both standards and samples were run in duplicate on a CFX96™ RT-PCR System with a C1000 Thermal Cycler (Bio-Rad, Hercules CA, USA). The Bio-Rad CFX Manager (version 1.6) software was used for calculation of threshold cycles (Ct)-values and melting curve analysis of amplified DNA. The average values of the duplicates were used for quantification of *H. heilmannii* s.s. DNA in the tissue samples.

### RNA preparation and gene expression

Total RNA, from the tissue samples, was purified using the RNeasy Mini Kit (Qiagen, Hilden, Germany), according to the manufacturer’s instructions. Purity of RNA was demonstrated by measuring the ratio of absorbance at 260 nm and 280 nm with NanoDrop which in all cases was approximately 2. The RNA concentration in each sample was adjusted to 1 μg/μL and cDNA was synthesized immediately after RNA purification using iScript™ cDNA Synthesis Kit (Bio-Rad). Aliquots of cDNA (1/5 dilution) were used as a template for quantitative RT-PCR for measuring gene expression. The mRNA expression levels of different cytokines (IL-1β, IL-5, IL-6, IL-10, IL-12p40, IL-17, IFN-γ and TNF-α), gastrin and H^+^/K^+^ ATPase were quantified. The housekeeping genes *GAPDH, β-actin* and *HPRT* were included as reference genes. Primer sequences are shown in Table 1 [23-28]. For all target genes and reference genes, the primer efficiencies were between 1.9 and 2.1. Reactions were performed in 10 μL volumes containing 1 μL cDNA, 0.05 μL of both primers, 3.9 μL HPLC water and 5 μL SensiMix™ SYBR No-ROX. The experimental protocol for PCR reaction (40 cycles) was performed on a CFX96™ RT-PCR System with a C1000 Thermal Cycler (Bio-Rad): denaturation for 15 min at 95 °C, followed by amplification cycles at 95 °C for 20 s, annealing at 60 °C for 30 s and extension at 73 °C for 30 s. Control reactions without the reverse transcriptase step were implemented to exclude DNA contamination of the RNA samples. No-template-control reaction mixtures were included and all samples were run in duplicate. The Ct-values were normalized to the geometric mean of the Ct-values from the 3 reference genes, after which normalized mRNA levels were calculated using the 2^-ΔΔCt^ method [29].

**Table 1:** Primer pairs.

	**Primers**	**Sequence (5’ → 3’)**
**Cytokines**	IL-1β FW^23^	GGC AGG TGG TAT CGC TCA TC
	IL-1β RV^23^	CAC CTT GGA TTT GAC TTC TA
	IL-5 FW	AGA GAA GTG TGG CGA GGA GAG ACG
	IL-5 RV	ACA GGG CAA TCC CTT CAT CGG
	IL-6 FW	CAA AGC CAG AGC CAT TCA GAG
	IL-6 RV	GCC ATT CCG TCT GTG ACT CCA GTT TCT CC
	IL-10 FW	GGT TGC CAA GCC TTA TCA GA
	IL-10 RV	GCT GCA TTC TGA GGG TCT TC
	IL-12p40 FW	GAC ACG ACC TCC ACC AAA GT
	IL-12p40 RV	CAT TCT GGG ACT GGA CCC TA
	IL-17 FW^23^	AGC TCC AGA GGC CCT CGG AC
	IL-17 RV^23^	AGG ACC AGG ATC TCT TGC TG
	IFN-γ FW^24^	CCA TGA ACG CTA CAC ACT GCA TC
	IFN-γ RV^24^	GAA GTA GAA AGA GAC AAT CTG G
	TNF-α FW^23^	GCT CCC CCA GAA GTC GGC G
	TNF-α RV^23^	CTT GGT GGT TGG GTA CGA CA
**Gastrin**	Gastrin FW^25^	GCC CTG GAA CCG CAA CA
	Gastrin RV^25^	TTC TTG GAC AGG TCT GCT TTG AA
**H**^**+**^**/K**^**+ **^**ATPase (parietal cells)**	ATP4b FW	GGG GGT AAC CTT GAG ACC TGA TG
	ATP4b RV	AAG AAG TAC CTT TCC GAC GTG CAG
**Reference genes**	GAPDH FW^26^	AAC GGC ACA GTC AAG GCT GAG AAC G
	GAPDH RV^26^	CAA CAT ACT CGG CAC CGG CAT CG
	HPRT FW^27^	CTC ATG GAC TGA TTA TGG ACA G
	HPRT RV^27^	AGC TGA GAG ATC ATC TCC ACC AAT
	β-actin FW^28^	CCA AGG CCA ACC GCG AGA TGA C
	β-actin RV^28^	AGG GTA CAT GGT GGT GCC GCC AGA C

Primer pairs used for measuring the mRNA expression levels of IL-1β, IL-5, IL-6, IL-10, IL-12p40, IL-17, IFN-γ, TNF-α, gastrin and H^+^/K^+^ ATPase are shown. The housekeeping genes *GAPDH, β-actin* and *HPRT* were included as reference genes.

### Statistical analysis

Normality and variance homogeneity of data were analyzed by using Shapiro-Wilk normality test and Levene’s test for homogeneity of variances. Gastritis scores, colonization capacity and ATPase and gastrin gene expression were compared between different infected groups and controls using Kruskall-Wallis analysis, followed by a Mann-Whitney *U* test. Cytokine expression and the number of Ki67-positive cells were analyzed by analysis of variance with a Bonferroni post hoc test. Differences were considered statistically significant at *p* ≤ 0.05. SPSS Statistics 21 software (IBM) was used for all analyses.

## Results

### Infection with virulent *H. heilmannii* s.s. strains induces an antrum-dominant chronic active gastritis

**Figure 1:**
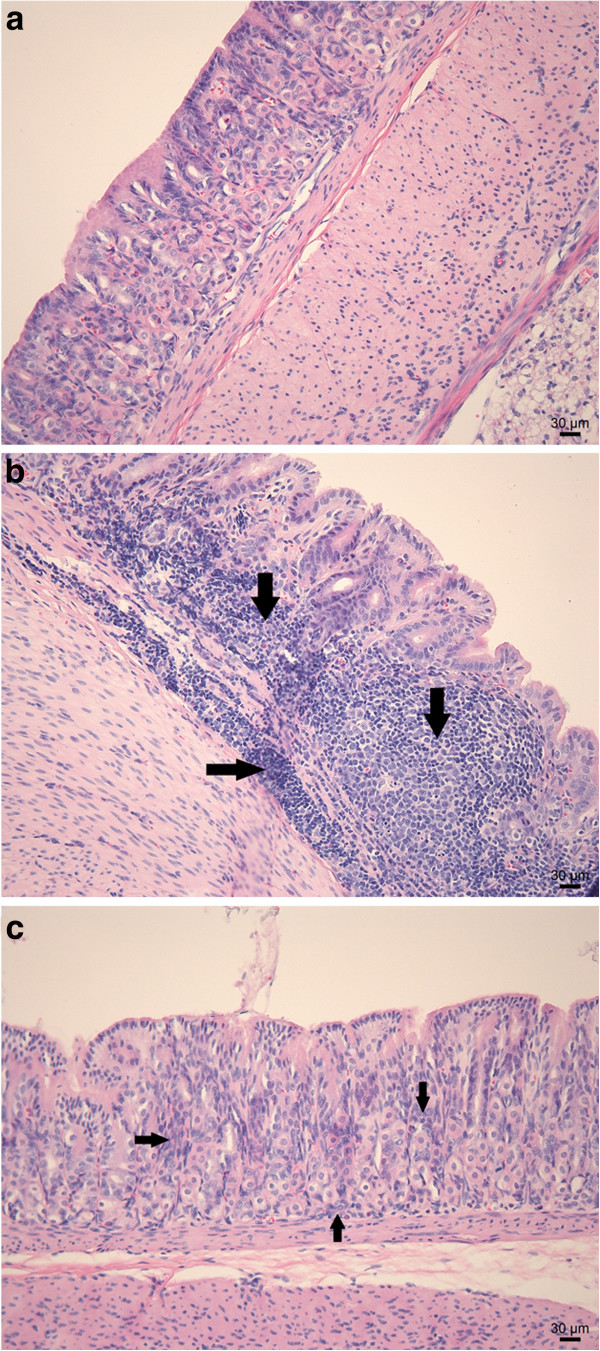
**H&E staining of the antrum of a gerbil stomach.** Normal histology of the antrum of a sham-inoculated negative control animal **(a)**. Explicit lymphocytic infiltration of the lamina propria and the submucosa with the formation of lymphoid follicles (arrows) in the antrum of a gerbil inoculated with *H. heilmannii* s.s. ASB1 **(b)**. Mild to absent lymphocytic infiltration (arrows) of the lamina propria in the antrum of a gerbil inoculated with *H. heilmannii* s.s. ASB7 **(c)**. Bar = 30 μm.

**Figure 2:**
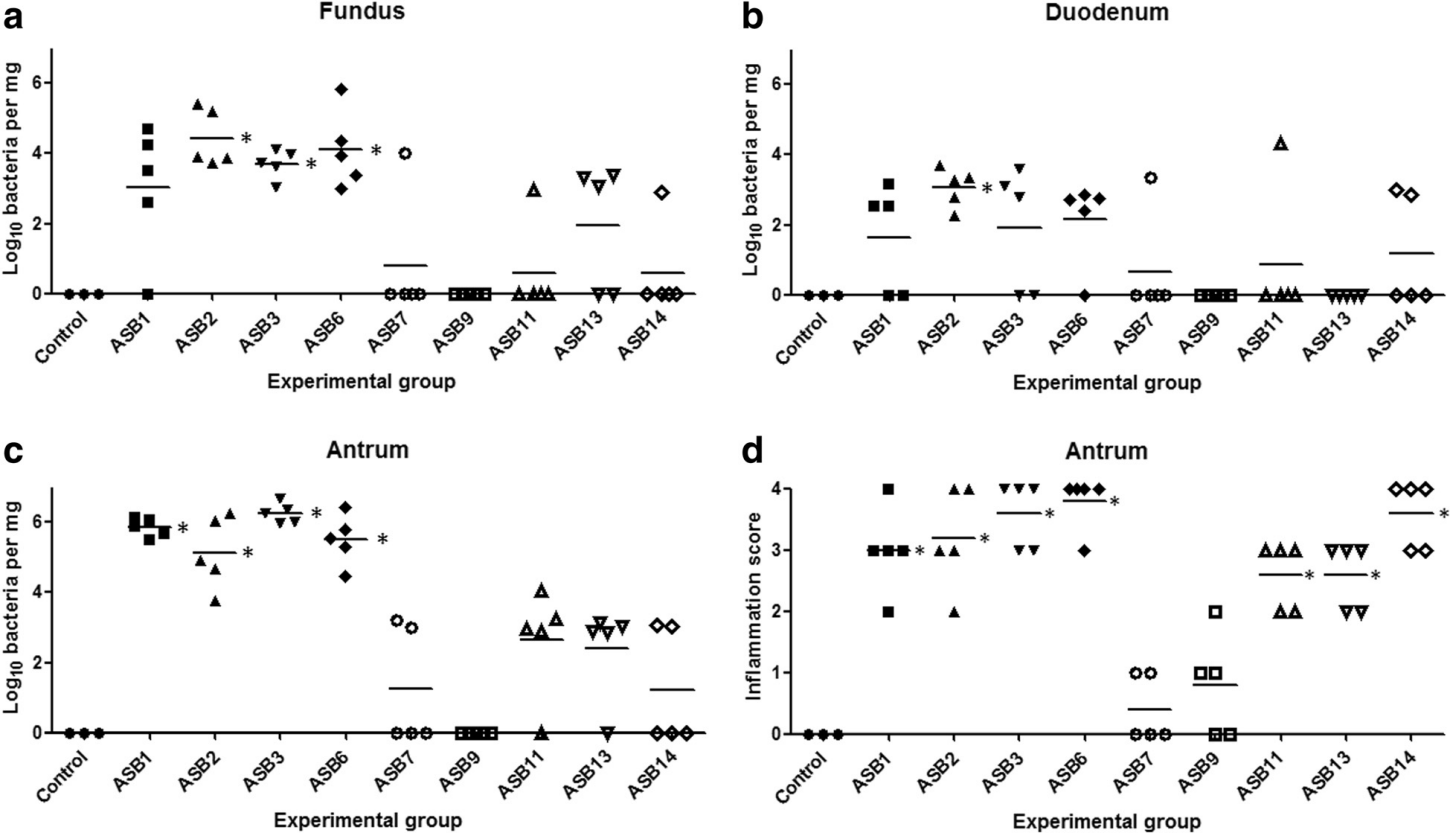
**Colonization capacity of *H. heilmannii *s.s. strains and gastric inflammation score after experimental infection.** Colonization capacity is shown as log_10_ values of *H. heilmannii* s.s. bacteria per mg tissue, detected with quantitative RT-PCR in the fundus **(a)** and the antrum **(c)** of the stomach and the duodenum **(b)**. Results below detection limit (log 2.39 bacteria per mg tissue) were set as 0. Antral inflammation **(d)** was scored on a scale of 0 to 4 (0: no infiltration with mononuclear and/or polymorphonuclear cells; 1: mild diffuse infiltration with mononuclear and/or polymorphonuclear cells or the presence of one small (50–200 cells) aggregate of inflammatory cells; 2: moderate diffuse infiltration with mononuclear and/or polymorphonuclear cells and/or the presence of 2–4 inflammatory aggregates; 3: marked diffuse infiltration with mononuclear and/or polymorphonuclear cells and/or the presence of at least five inflammatory aggregates; 4: diffuse infiltration of large regions with large aggregates of mononuclear and/or polymorphonuclear cells).Individual gerbils are depicted as figures around the mean (lines). Statistical significant differences compared to control animals are indicated by * (Mann-Whitney *U* test, *p* < 0.05).

The stomach of all control animals showed a normal histomorphology (Figure 1a). Inflammation in the stomach of gerbils infected with *H. heilmannii* s.s. strains ASB1, ASB2, ASB3, ASB6, ASB11, ASB13 and ASB14 was marked by a chronic active gastritis with formation of lymphocytic aggregates in the lamina propria and submucosa of the antrum of the stomach (Figure 1b). The mucosal thickness was slightly increased and only few neutrophils were detected. In contrast, *H. heilmannii* s.s. strains ASB7 and ASB9 did not cause explicit antral inflammation and only a mild increase in lymphocytic cell number was observed in the lamina propria of the antrum of the stomach (Figure 1c). In all *H. heilmannii* s.s.-infected gerbils, only limited signs of inflammation were detected in the fundus of the stomach (Additional file 1). The antral inflammation scores of each individual animal are shown in Figure 2d. A statistically significant difference between inflammation scores for gerbils inoculated with ASB1, ASB2, ASB3, ASB6, ASB11, ASB13 and ASB14 compared with the control group was demonstrated (Mann-Whitney *U* test, *p* < 0.05, Figure 2d).

### Colonization capacity of *H. heilmannii* s.s. strains in the stomach and duodenum

Detection of *H. heilmannii* s.s. DNA with quantitative RT-PCR at 9 weeks post-infection revealed high-level colonization of ASB1, ASB2, ASB3 and ASB6 in the stomach (Figure 2a-c). In contrast, colonization of ASB7, ASB11, ASB13 and ASB14 was more restricted while ASB9 was not detected in the stomach (Figure 2a-c). In general, the colonization capacity in the fundus (Figure 2a) was lower than in the antrum (Figure 2c) for all strains tested and the lowest number of bacteria was detected in the duodenum (Figure 2b). In addition, a clear association was seen between the colonization capacity of the *H. heilmannii* s.s. strains and the gastric inflammation scores in the antrum of the stomach (Figure 2c-d).

### Virulent *H. heilmannii* s.s. strains cause gastric antral epithelial cell proliferation

**Figure 3:**
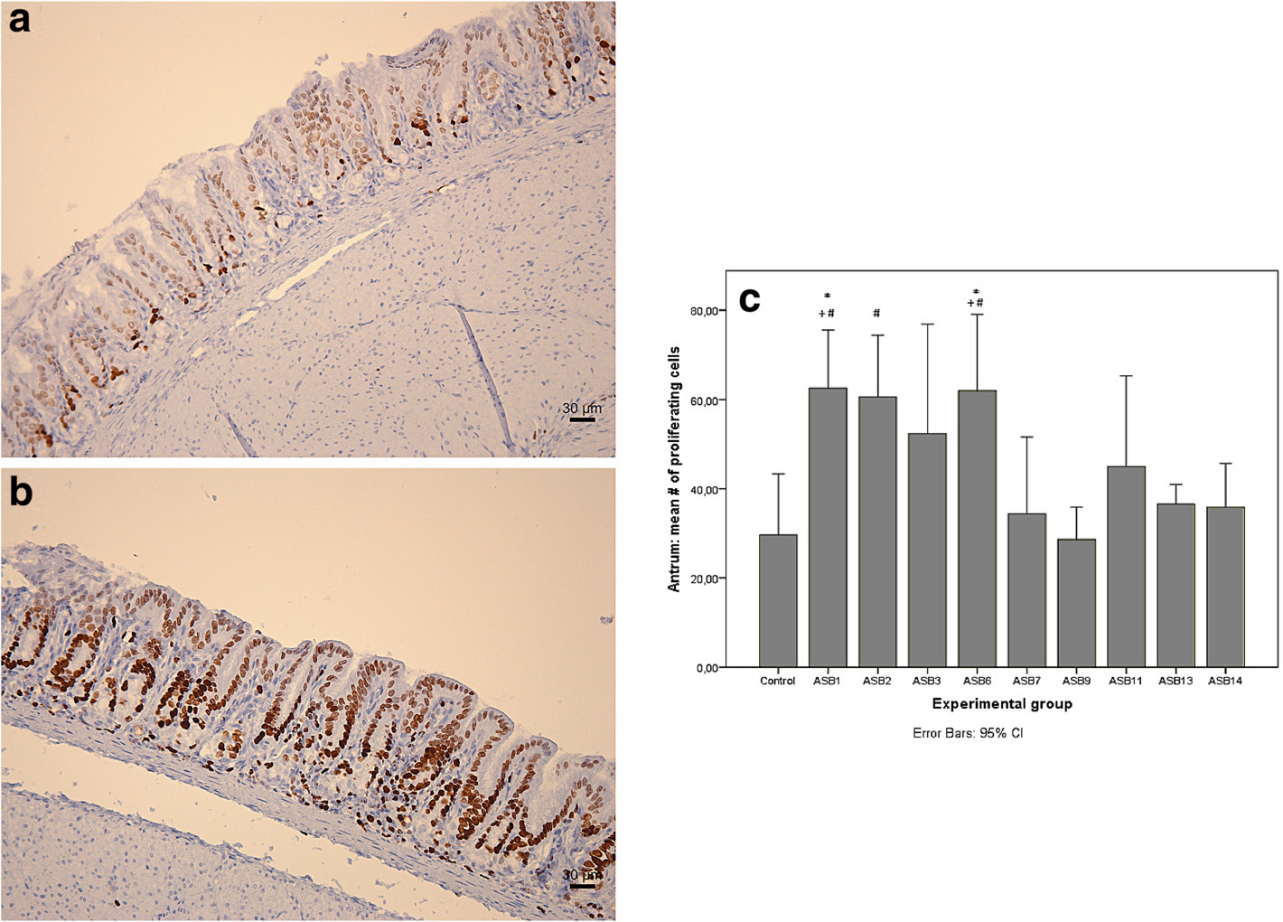
**Gastric antral epithelial cell proliferation.** Ki67 staining of the antrum of a gerbil inoculated with *H. heilmannii* s.s. ASB1 **(b)** showing a higher number of proliferating epithelial cells compared to a sham-inoculated negative control animal **(a)**. The rate of epithelial cell proliferation was determined by counting Ki67-positive epithelial cells in 5 randomly chosen High Power Fields at the level of the gastric pits (magnification: 400×) in the antrum of the gerbil stomach **(c)**. The mean numbers of Ki67-positive cells are shown in each experimental group. Significant differences between *H. heilmannii* s.s.-inoculated and control animals are indicated by * (ANOVA, *p* < 0.05). Significant differences in comparison with ASB7 and ASB9 inoculated groups are indicated by + and # respectively (ANOVA, *p* < 0.05).

Results of the gastric epithelial cell proliferation scoring in the antrum of the stomach are shown in Figure 3c. Significantly higher numbers of Ki67-positive proliferating epithelial cells were seen in the antrum of ASB1- and ASB6-infected gerbils, compared to the control group (ANOVA, *p* < 0.05, Figure 3a-b). Numbers of Ki67-positive cells were moderately increased in ASB2-, ASB3-, ASB11-, ASB13- and ASB14-infected gerbils, although not statistically significant. *H. heilmannii* s.s. strains ASB7 and ASB9 did not cause an increase of gastric epithelial cell proliferation. In addition, significantly higher numbers of proliferating epithelial cells were demonstrated in the antrum of ASB1-, ASB2-, and ASB6-infected gerbils, compared to gerbils infected with ASB7 and ASB9 (ANOVA, *p* < 0.05).

In the fundus of all *H. heilmannii* s.s.-infected gerbils, the epithelial cell proliferation rate was not significantly higher compared to the control animals (Additional file 2).

### Cytokine gene expression in the stomach in response to *H. heilmannii* s.s. infection

**Figure 4:**
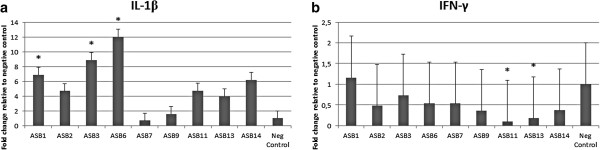
**mRNA expression levels of cytokines IL-1β and IFN-γ in the antrum of the stomach.** Cytokine mRNA expression levels in the fundus and antrum of the stomach were examined by quantitative RT-PCR. Expression levels of IL-1β **(a)** and IFN-γ **(b)** in the antrum are shown. Data are presented as the fold change in gene expression normalized to 3 reference genes and relative to the negative control group which is considered as 1. Data are shown as means + standard deviation. Significant differences in expression level between inoculated groups and negative control group are indicated by * *p* < 0.05 (ANOVA).

The local host immune response towards *H. heilmannii* s.s. infection was characterized by measuring the mRNA expression level of IFN-γ, IL-1β, IL-5, IL-6, IL-10, IL-12p40, IL-17 and TNF-α in the stomach of the gerbils. Results are shown in Table 2 and in Figure 4.

**Table 2:** Statistical analysis of mRNA expression levels.

			**Antrum**				**Fundus**	
	**IL-1β**		**IFN-γ**		**H**^**+**^**/K**^**+ **^**ATPase**		**Gastrin**	
	**Mean Ct-Ctref**^**a**^	***p*-value**^**b**^	**Mean Ct-Ctref**	***p*-value**	**Mean Ct-Ctref**	***p*-value**	**Mean Ct-Ctref**	***p*-value**
**ASB1**	6.92 ± 0.29 *	0.031	6.87 ± 0.60	1.000	3.70 ± 2.11 *	0.050	7.20 ± 1.68	0.101
**ASB2**	7.48 ± 1.42	0.231	8.15 ± 1.26	1.000	2.42 ± 5.01	0.513	5.94 ± 2.49 *	0.050
**ASB3**	6.55 ± 0.80 *	0.008	7.54 ± 0.37	1.000	3.49 ± 2.28 *	0.050	7.71 ± 1.17	0.101
**ASB6**	6.12 ± 0.67 *	0.001	7.99 ± 0.90	1.000	2.23 ± 1.99 *	0.050	5.33 ± 2.39 *	0.025
**ASB7**	10.25 ± 0.37	1.000	7.98 ± 0.52	1.000	0.45 ± 2.51	0.724	7.12 ± 1.54	0.180
**ASB9**	9.03 ± 2.54	1.000	8.55 ± 0.71	1.000	0.79 ± 0.03	0.564	8.11 ± 1.58	1.000
**ASB11**	7.47 ± 0.15	0.499	10.45 ± 0.95 *	0.004	1.32 ± 1.45	0.248	7.67 ± 1.25	0.289
**ASB13**	7.72 ± 1.17	0.523	9.54 ± 1.55 *	0.044	3.80 ± 0.34	0.083	8.10 ± 0.93	0.456
**ASB14**	7.07 ± 0.52	0.054	8.51 ± 0.97	1.000	3.81 ± 3.11	0.127	7.77 ± 2.96	0.881
**Negative Control**	9.71 ± 1.13	-	7.08 ± 0.65	-	0.15 ± 1.82	-	8.70 ± 0.62	-

Shown is the statistical analysis of IL-1β, IFN-γ and H^+^/K^+^ ATPase mRNA expression in the antrum and gastrin mRNA expression in the fundus of the gerbil stomach at 9 weeks after experimental infection. Cytokine expression was analyzed by analysis of variance with a Bonferroni post hoc test. H^+^/K^+^ ATPase and gastrin gene expression were compared between different infected groups and controls using Kruskall-Wallis analysis, followed by a Mann-Whitney *U* test. Differences were considered statistically significant at *p* ≤ 0.05. SPSS Statistics 21 software (IBM) was used for all analyses.

^a^ Mean Ct-Ctref: For each experimental group, the mean of the normalized Ct-values ± standard deviation are shown.

^b^*p*-value: the exact *p*-values are given.

* Statistically significant differences compared to the uninfected control group (*p* ≤ 0.05).

The pro-inflammatory cytokine IL-1β is a potent inhibitor of gastric acid secretion [30] and plays a role in the acute phase of inflammation [31]. Expression of IL-1β was up-regulated in the antrum of the stomach of gerbils infected with *H. heilmannii* s.s. ASB1, ASB2, ASB3, ASB6, ASB11, ASB13 and ASB14, compared to the negative control animals (Figure 4a). For gerbils inoculated with ASB7 and ASB9, no up-regulation of IL-1β was seen.

The Th1 cytokine IFN-γ, a signature marker of the Th1-polarized response [24,32], exhibited a decreased expression in the antrum of gerbils infected with ASB11 and ASB13 (Figure 4b), compared to the control animals.

No significant differences in expression between infected and sham-inoculated gerbils could be observed for IL-5, IL-6, IL-10, IL-12p40, IL-17 and TNF-α.

### Parietal cell H^+^/K^+^ ATPase mRNA expression is down-regulated in response to colonization with *H. heilmannii* s.s

**Figure 5:**
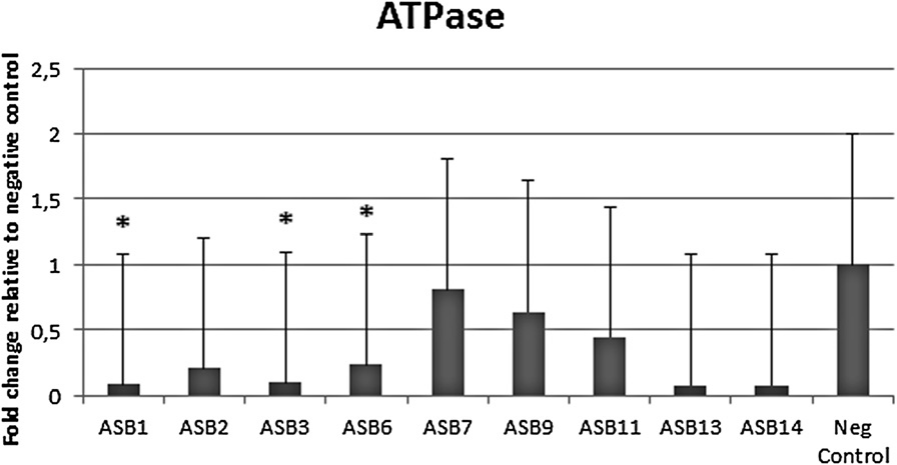
**mRNA expression level of H**^**+**^**/K**^**+ **^**ATPase in the antrum of the stomach.** Hydrogen potassium ATPase mRNA expression level in the stomach was examined by quantitative RT-PCR. H^+^/K^+^ ATPase mRNA expression level in the antrum is shown. Data are presented as the fold change in gene expression normalized to 3 reference genes and relative to the negative control group which is considered as 1. Data are shown as means + standard deviation. Significant differences in expression level between inoculated groups and negative control group are indicated by * *p* ≤ 0.05 (Mann-Whitney *U* test).

No clear loss of parietal cells could be visualized by immunohistochemical staining in the fundus and the antrum of the *H. heilmannii* s.s.-infected gerbils compared to the uninfected controls (data not shown). However, quantitative RT-PCR showed a clear decrease in the expression of gastric H^+^/K^+^ ATPase in the antrum of the gerbils infected with ASB1, ASB3 and ASB6 (Figure 5 and Table 2). Compared to the control animals with mRNA expression levels set to 1.0, the mean relative expression was 0.09 ± 2.11 for ASB1-, 0.10 ± 2.28 for ASB3- and 0.24 ± 1.99 for ASB6-infected gerbils, respectively. No significant change in expression was seen in the fundus of the *H. heilmannii* s.s.-infected gerbils.

### Virulent *H. heilmannii* s.s. strains induce increased gastrin expression in the fundus

**Figure 6:**
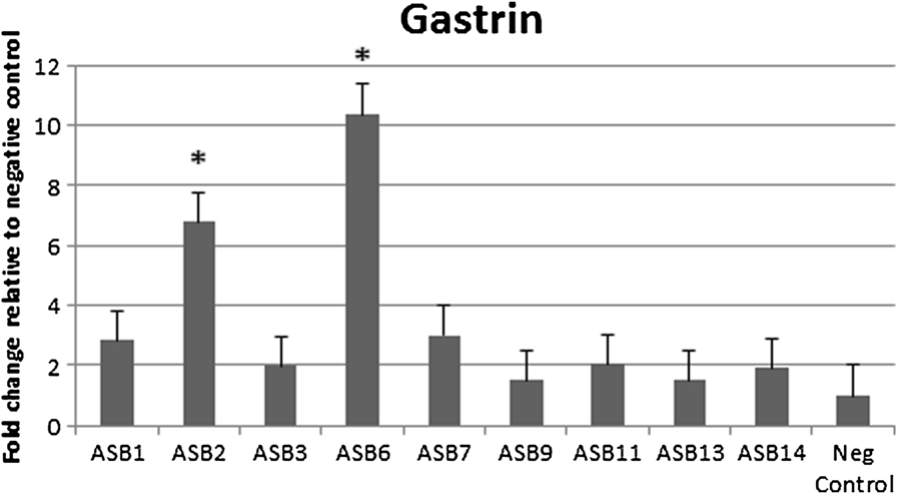
**mRNA expression level of gastrin in the fundus of the stomach.** Gastrin mRNA expression level in the stomach was examined by quantitative RT-PCR. Shown is the expression level in the fundus. Data are presented as the fold change in gene expression normalized to 3 reference genes and relative to the negative control group which is considered as 1. Data are shown as means + standard deviation. Significant differences in expression level between inoculated groups and negative control group are indicated by * *p* ≤ 0.05 (Mann-Whitney *U* test).

The peptide hormone gastrin stimulates the secretion of gastric acid by parietal cells. A disturbance in its expression may lead to hypergastrinemia. The expression of gastrin was highly up-regulated in the fundus of gerbils infected with ASB2 and ASB6 at 9 weeks post-infection (Figure 6 and Table 2). Compared to control animals, the mean relative expression was 6.79 ± 2.49 for ASB2- and 10.35 ± 2.39 for ASB6-infected gerbils. In the antrum of the stomach, no up-regulation of gastrin expression was detected.

## Discussion

At 9 weeks post inoculation, a chronic active gastritis in the antrum of the stomach was observed in gerbils experimentally infected with 7 out of 9 *H. heilmannii* s.s. strains tested in this study (ASB1, ASB2, ASB3, ASB6, ASB11, ASB13 and ASB14). The lamina propria and submucosa were massively infiltrated with lymphocytes, resulting in the formation of lymphoid follicles. The *H. heilmannii* s.s. strains were mainly detected in the antrum and to a lesser extent in the fundus and the duodenum. In humans infected with NHPH, colonization and inflammation also mainly occur in the antrum of the stomach [5,33-35]. This confirms that Mongolian gerbils are an appropriate model to study *H. heilmannii* s.s. infections in humans, as has also been shown for *H. suis*[19] and *H. pylori*[20,21]. No inflammation was seen in the fundus of the stomach of the *H. heilmannii* s.s.-infected gerbils. Wiedemann et al. [25] demonstrated that in *H. pylori*-infected Mongolian gerbils a fundus-dominant gastritis is dependent on a functional Cag pathogenicity island. The *H. heilmannii* s.s. genome lacks a Cag pathogenicity island [36], which might explain the antrum-dominant gastritis and the absence of inflammation in the fundus.

The highest number of colonizing bacteria was seen in the antrum of gerbils inoculated with ASB1, ASB2, ASB3 and ASB6. The colonization capacity of ASB7, ASB11, ASB13 and ASB14 was more restricted. ASB9 could not be detected in the stomach nor the duodenum of the gerbils at 9 weeks after experimental infection, which might indicate that the infection was cleared within 9 weeks, or that ASB9 was not able to colonize the gastric mucosa. Also, *H. heilmannii* s.s. strains ASB7 and ASB9 did not cause explicit lymphocytic inflammation or gastric lesions. These results indicate that the capacity of *H. heilmannii* s.s. to colonize the stomach and to cause inflammation and lesions is strain-dependent.

The risk to develop MALT lymphoma is considered to be higher in humans infected with NHPH than in *H. pylori-*infected patients [5,37-39] and MALT lymphoma-like lesions have been demonstrated in the stomach of Mongolian gerbils colonized with *H. suis* for 8 months [19]. MALT lymphoma is characterized by an extensive proliferation of B-lymphocytes which may be dependent on Th2-type cytokines. Indeed, experimental *H. suis* infections in mouse models have been shown to evoke a Th2-polarized response [19,40]. Surprisingly, in the present study, there was no up-regulation of the Th2-cytokine IL-5 in the stomach of the *H. heilmannii* s.s.-colonized gerbils at 9 weeks post inoculation. It remains to be determined if long-term colonization of Mongolian gerbils with *H. heilmannii* s.s. would induce a prolonged Th2-polarized response eventually resulting in MALT lymphoma-like lesions.

In the present study, mRNA levels of the pro-inflammatory cytokine IL-1β were up-regulated in the antrum of gerbils suffering from gastritis. Up-regulation of this cytokine has also been demonstrated in the stomach of *H. pylori*-infected gerbils [25,31]. Another cytokine playing a role in gastric inflammation in *H. pylori*-infected Mongolian gerbils is the Th17 cytokine IL-17 [23]. *H. suis*-infection in mouse models has been shown to induce a predominant Th17 response as well [40]. In our study, there was no significant up-regulation of IL-17 at 9 weeks post-infection. Since IL-17 has been shown to be a key regulator of neutrophil infiltration [19,41,42], the absence of a Th17 response might explain the low number of infiltrating neutrophils in the antral mucosa of the gerbils with gastritis. Examination of samples taken at other time points after infection will be needed to elucidate the importance of IL-17 in the maintenance and regulation of chronic gastric inflammation during an *H. heilmannii* s.s. infection.

A major difference with *H. pylori* infections is the absence of an up-regulation of IFN-γ in the stomach of gerbils infected with *H. heilmannii* s.s. An *H. pylori* infection in mice, gerbils and humans is indeed accompanied by a Th1-polarized response, characterized by a strong increase of IFN-γ [25,31,43,44]. In contrast, expression levels of IFN-γ were even lower in the stomach of gerbils infected with *H. heilmannii* s.s. strains ASB11 and ASB13 compared to sham-inoculated control gerbils. Absence of a Th1-polarized response has also been described for *H. suis* infection in mice [40]. This demonstrates that the pathogenesis of gastric NHPH infections is not identical to that of an *H. pylori* infection.

Gastric acid secretion is mediated by the gastric hydrogen potassium ATPase (H^+^/K^+^ ATPase), that functions as a proton pump in the gastric acid-secreting parietal cells [45]. Although acid-secreting parietal cells are characteristic for the fundic epithelium, they are also observed in the gastric antrum of the Mongolian gerbil, albeit to a lesser extent [19]. In the present study, a reduction in the antral expression of H^+^/K^+^ ATPase was detected for 3 *H. heilmannii* s.s. strains (ASB1, ASB3 and ASB6), suggesting reduced gastric acid secretion which might lead to antral mucosal atrophy. Mucosal atrophy of the antrum has also been described in Mongolian gerbils infected with *H. pylori* and with *H. suis*[19,25]. However, the relevance of a reduced antral H^+^/K^+^ ATPase expression for the physiology of the stomach remains unclear, since the majority of parietal cells are located in the fundus of the stomach.

The peptide hormone gastrin is released by G-cells mainly in the antrum of the stomach in response to food intake and stimulates the secretion of gastric acid by parietal cells [46]. *H. pylori* infection in human patients and animal models is commonly associated with increased gastrin levels and is considered to be a reaction to the *H. pylori-*induced hypochlorhydria [47,48]. In an attempt to repair acid homeostasis, gastrin stimulates histamine release from enterochromaffin-like (ECL) cells, inducing acid secretion [49-51]. Moreover, IL-1β, which is up-regulated after a *H. pylori* infection, stimulates gastrin release from antral G-cells and inhibits antral D-cells to express somatostatin, an inhibitor of gastrin-stimulated acid secretion [25,52]. This does, however, not result in increased production of hydrochloric acid due to a modulating effect of IL-1β on the *H. pylori*-mediated H^+^/K^+^ ATPase α-subunit promoter inhibition, contributing to reduced parietal cell H^+^/K^+^ ATPase gene and protein expression and thus to hypochlorhydria [53]. As mentioned above, IL-1β was also up-regulated in the stomach of our gerbils with gastritis and in the present study, gastrin mRNA was up-regulated in the fundus of gerbils inoculated with strains ASB2 and ASB6. While G-cells were most abundant in the antrum of the stomach, some could also be seen at the edge of the fundus of the *H. heilmannii* s.s.-infected gerbils, in the transition zone between fundus and antrum (Additional file 3). It should be noted that an increased level of gastrin mRNA does not necessarily mean a higher level of active gastrin hormone, as the translated precursor protein progastrin has to be processed by posttranslational modifications into its active form gastrin [46]. In *H. pylori*-infected gerbils, gastrin levels started to increase only after 16 weeks of infection and mainly in antral tissue [25]. Further studies, measuring the levels of IL-1β, gastrin, histamine and somatostatin after long term experimental infection, are necessary to obtain additional insights into the influence of *H. heilmannii* s.s. on gastric homeostasis.

In conclusion and taking together the results of histopathology, antral epithelial cell proliferation, colonization capacity and cytokine, H^+^/K^+^ ATPase and gastrin expression, the present experimental infection studies in Mongolian gerbils indicate variation in bacterium-host interactions and virulence between different *H. heilmannii* s.s. isolates. Since the Mongolian gerbil model is considered to be a good model for human *Helicobacter*-induced pathology [19-21], this strain variation is most probably also relevant for human infections with this microorganism and might be important for infections in the natural hosts of *H. heilmannii* s.s., dogs and cats, as well. Future research is necessary to determine if the variation in virulence can be explained by specific virulence genes present in highly virulent strains, or by differences in expression of such genes between highly virulent and less virulent strains.

## Competing interests

The authors declare that they have no competing interests.

## Authors’ contributions

MJ participated in the design of the study, performed the experiments, analyzed the data and drafted the manuscript. CB participated in the experiments. BF and RD participated in the design of the study and edited the manuscript. FH and AS coordinated the study, participated in the design of the study, helped to interpret the results and edited the manuscript. All authors read and approved the final manuscript.

## Authors’ information

Freddy Haesebrouck and Annemieke Smet shared senior authorship.

## Supplementary Material

Additional file 1 **H&E staining of the fundus of a gerbil stomach.** Normal histology of the fundus of a sham-inoculated negative control animal (a). Comparable normal histology of the fundus of a gerbil inoculated with *H. heilmannii* s.s. ASB1 (b) and *H. heilmannii* s.s. ASB7 (c). Bar = 30 μm. Click here for file

Additional file 2 **Ki67 staining of the fundus of a gerbil stomach.** Ki67 staining of the fundus of a sham-inoculated negative control animal (a) and of a gerbil inoculated with *H. heilmannii* s.s. ASB1 (b) showing an equal number of proliferating epithelial cells. Click here for file

Additional file 3 **Gastrin staining of the fundus of a gerbil stomach.** The presence of G-cells in the fundus of the stomach was analyzed by immunohistochemical staining using a polyclonal rabbit anti-gastrin-17 antibody (1/800 dilution, Code No A0568, DAKO A/S, Denmark). Some G-cells are located in the transition zone between the fundus and the antrum in gerbils inoculated with *H. heilmannii* s.s. ASB2 (a) or with *H. heilmannii* s.s. ASB6 (b). Click here for file
