# Supplementary figures and images for: Diversity in bacterium-host interactions within the species Helicobacter heilmannii sensu stricto
Source: Vet Res. 2013 Jul 29;44(1):65. doi: 10.1186/1297-9716-44-65 (PMC3750284; doi:10.1186/1297-9716-44-65)

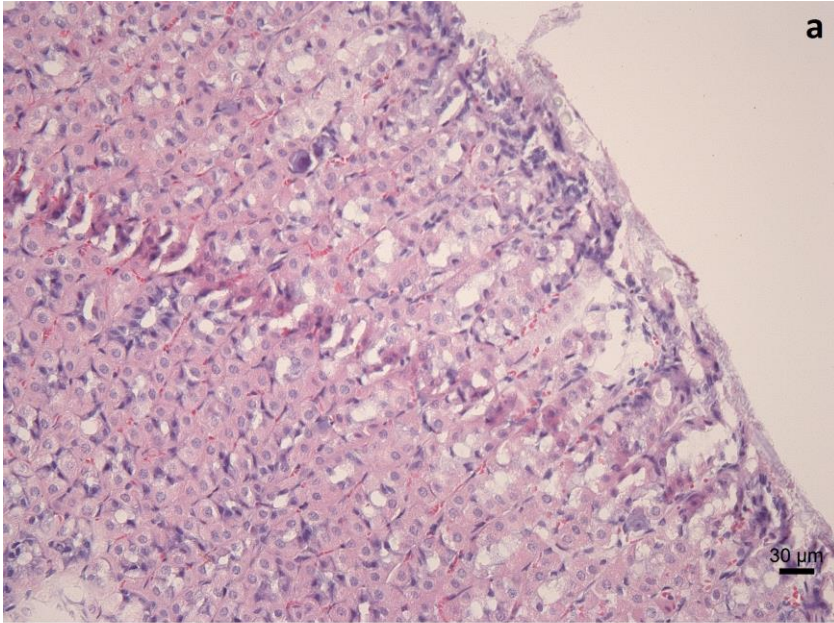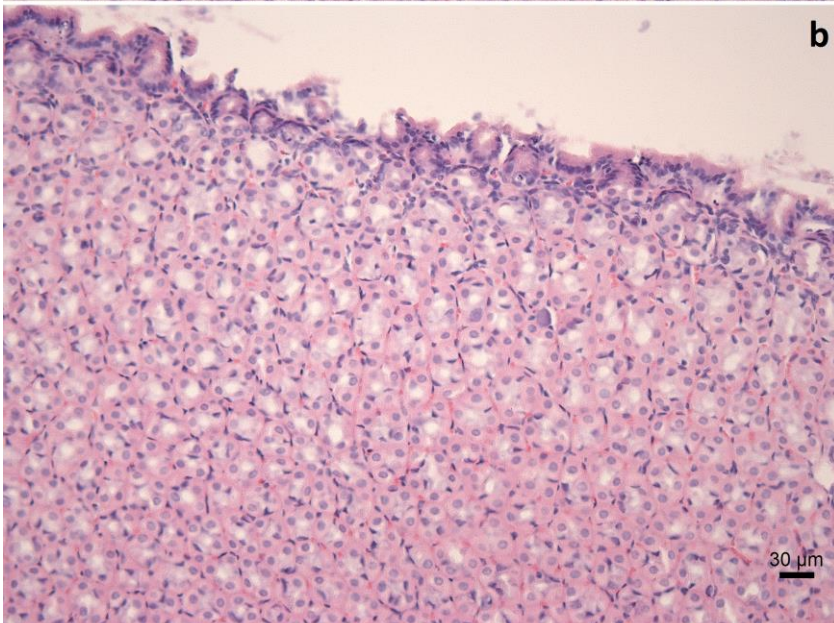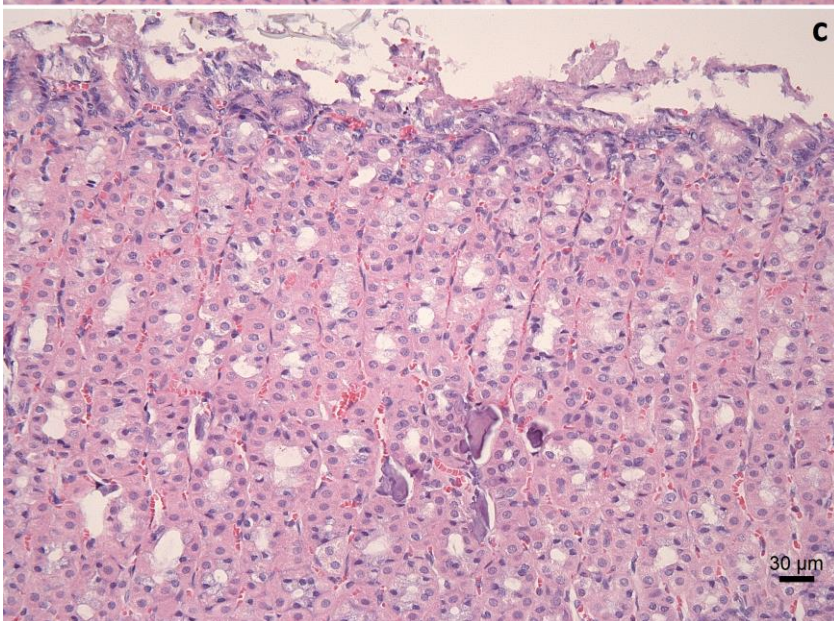

Supplement: Additional file 1 — H&E staining of the fundus of a gerbil stomach. Normal histology of the fundus of a sham-inoculated negative control animal (a). Comparable normal histology of the fundus of a gerbil inoculated with H. heilmannii s.s. ASB1 (b) and H. heilmannii s.s. ASB7 (c). Bar = 30 μm. [file 1297-9716-44-65-S1.pdf]

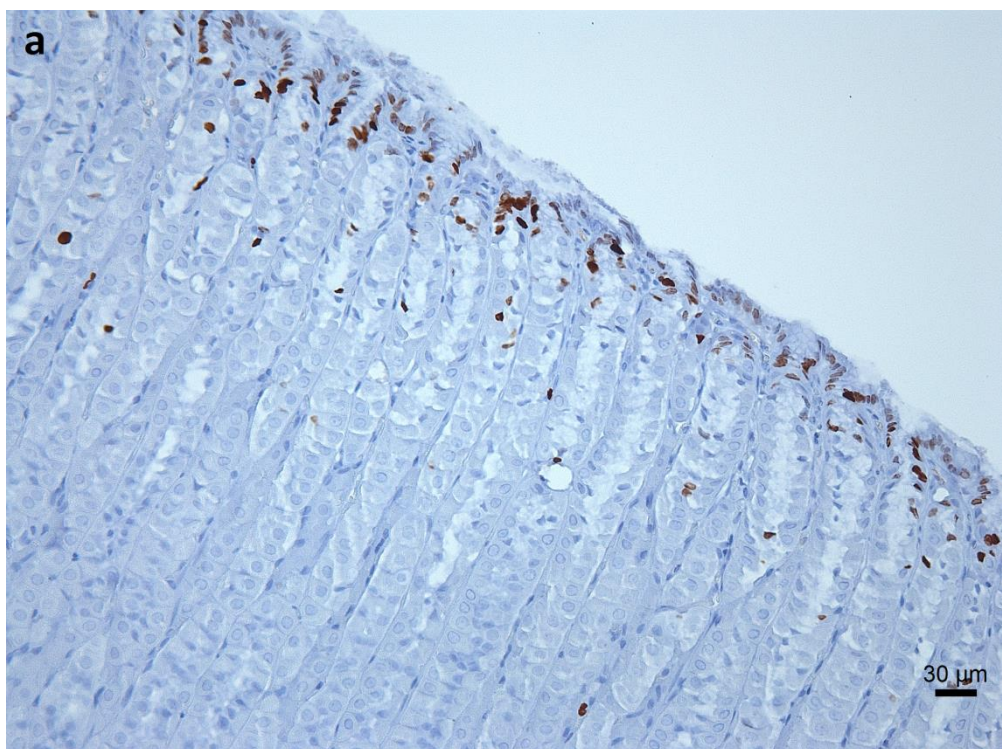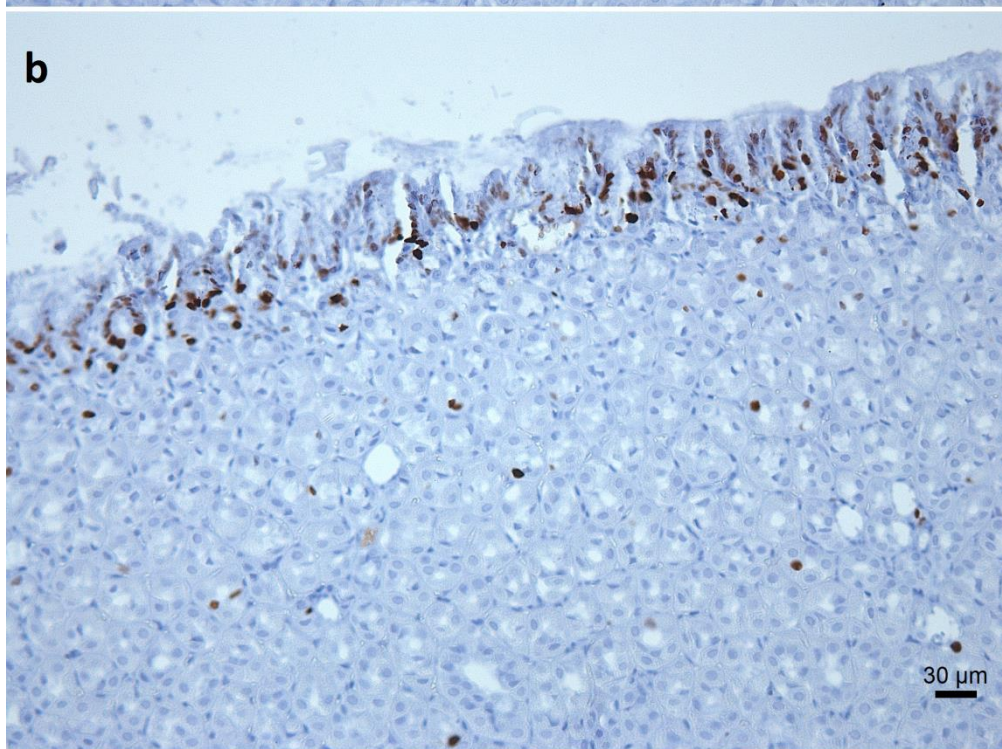

Supplement: Additional file 2 — Ki67 staining of the fundus of a gerbil stomach. Ki67 staining of the fundus of a sham-inoculated negative control animal (a) and of a gerbil inoculated with H. heilmannii s.s. ASB1 (b) showing an equal number of proliferating epithelial cells. [file 1297-9716-44-65-S2.pdf]

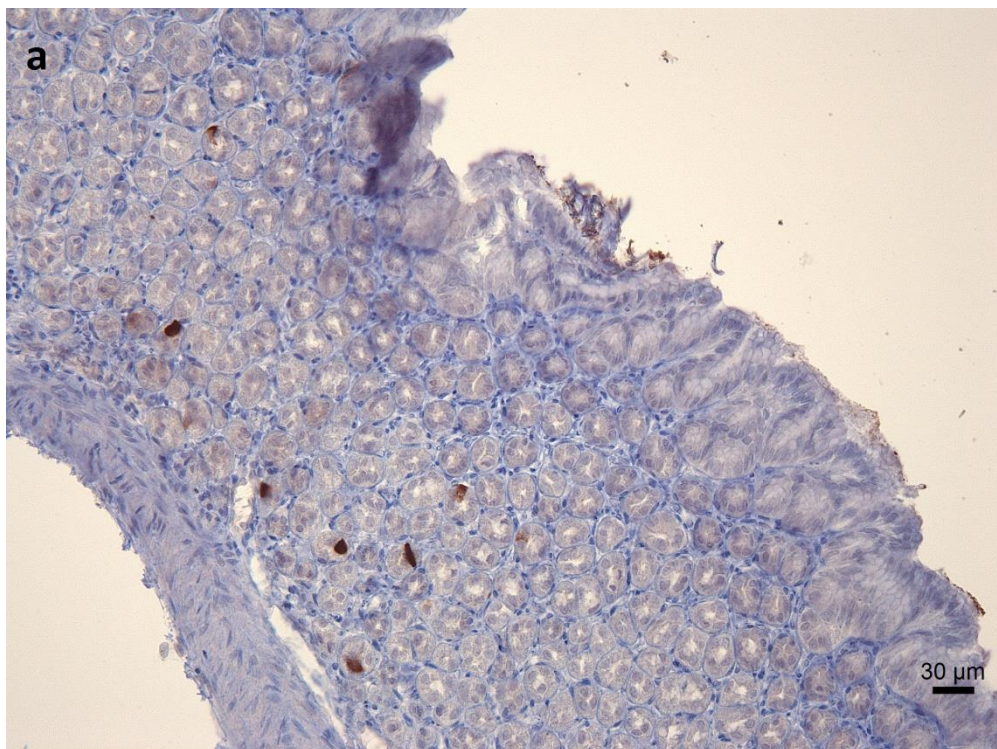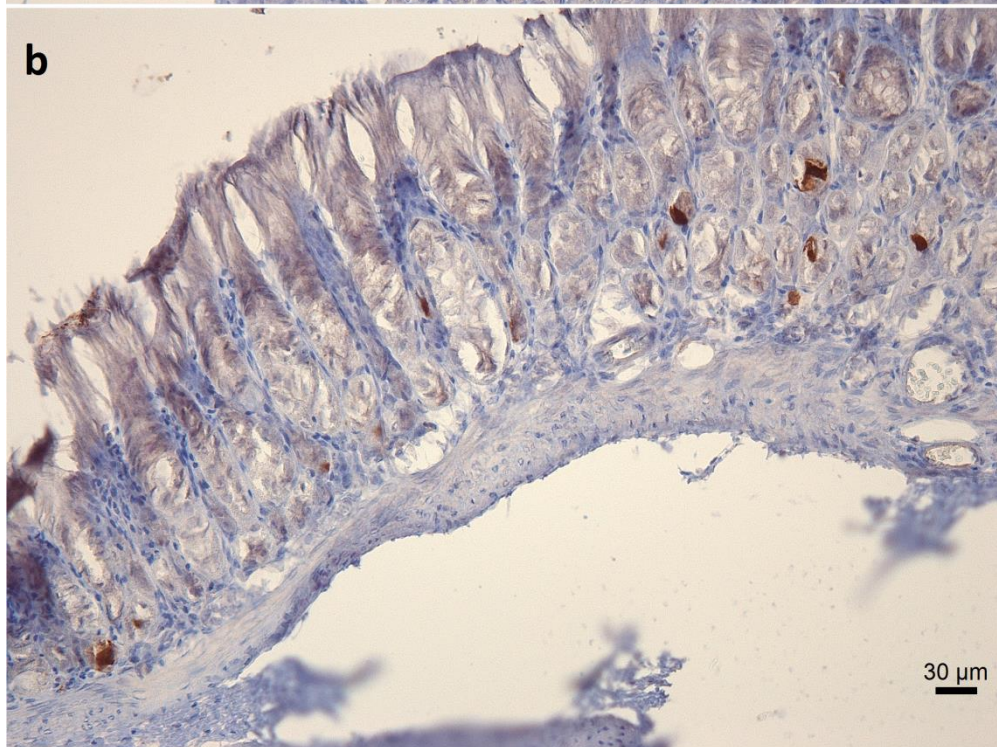

Supplement: Additional file 3 — Gastrin staining of the fundus of a gerbil stomach. The presence of G-cells in the fundus of the stomach was analyzed by immunohistochemical staining using a polyclonal rabbit anti-gastrin-17 antibody (1/800 dilution, Code No A0568, DAKO A/S, Denmark). Some G-cells are located in the transition zone between the fundus and the antrum in gerbils inoculated with H. heilmannii s.s. ASB2 (a) or with H. heilmannii s.s. ASB6 (b). [file 1297-9716-44-65-S3.pdf]
